# Performance of HCV Antigen Testing for the Diagnosis and Monitoring of Antiviral Treatment: A Systematic Review and Meta-Analysis

**DOI:** 10.1155/2022/7348755

**Published:** 2022-01-04

**Authors:** Geane Lopes Flores, Jurema Corrêa Mota, Larissa Tropiano da Silva Andrade, Renata Serrano Lopes, Francisco Inácio Bastos, Livia Melo Villar

**Affiliations:** ^1^Laboratory of Viral Hepatitis, Oswaldo Cruz Institute, FIOCRUZ, Rio de Janeiro, Brazil; ^2^Institute of Communication and Information on Science and Technology in Health, FIOCRUZ, Rio de Janeiro, Brazil

## Abstract

**Background and Aims:**

Active hepatitis C virus (HCV) infection is based on the detection of HCV RNA that it is effective but presents high cost and the need to hire trained personnel. This systematic review and meta-analysis is aimed at evaluating the diagnostic accuracy of HCV Ag testing to identify HCV cases and to monitor antiviral treatment including DAA treatment.

**Methods:**

The studies were identified through a search in PubMed, Lilacs, and Scopus from 1990 through March 31, 2020. Cohort, cross-sectional, and randomized controlled trials were included. Two independent reviewers extracted data and assessed quality using an adapted Quality Assessment of Diagnostic Accuracy Studies (QUADAS-2) tool. Our primary outcome was to determine the accuracy of HCV Ag detection for the diagnosis, which we estimated using random-effects meta-analysis.

**Results:**

Of 3,062 articles identified, 54 met our eligibility criteria. The studies described cohorts from 20 countries, including 14,286 individuals with chronic HCV individuals. Studies for ECLIA technology demonstrated highest quality compared to studies that used ELISA. The pooled sensitivity and specificity (95% CI) for HCV Ag detection of active HCV infection were 98.82% (95%CI = 98.04%; 99.30%) and 98.95% (95%CI = 97.84%; 99.49%), respectively. High concordance was found between HCV Ag testing and HCV RNA detection 89.7% and 95% to evaluate antiviral treatment.

**Conclusions:**

According to our findings, HCV Ag testing could be useful to identify HCV active cases in low-resource areas. For antiviral treatment, HCV Ag testing will be useful at the end of treatment.

## 1. Introduction

Diagnosis of hepatitis C virus (HCV) infection is primarily performed through the detection of the HCV antibodies (anti-HCV), although this test does not differentiate past and current infections. Further information requires HCV-RNA testing. The execution of the latter is difficult due to the need to hire trained personnel, the use of specialized equipment, and the high cost of reagents [1, 2].

Early diagnosis of HCV is important to identify acute and chronic cases and to initiate and monitor therapeutic strategy. Due to this, the identification of the infection in its acute phase is very important for a good clinical prognosis [3–5]. From a complementary point of view, considering most infections are asymptomatic and the fast-viral replication during this phase, it is key to detect acute infections in a prompt and reliable way. Mathematical modeling has highlighted the relevance of acute asymptomatic infections for the overall dynamics of HCV [6].

To reduce costs and improve the access, some studies have used HCV Ag in serum or plasma to diagnose hepatitis C infection. HCV Ag appears approximately 2 to 3 weeks after the contact to virus, almost simultaneously as HCV RNA [7]. HCV antigen (HCV Ag) detection test was used to diagnose HCV in several cases, principally among risk population, immunocompromised individuals and patients under hemodialysis due to its low cost and the shorter time required to get its results [8, 9]. HCV Ag test was also used to monitor antiviral response to PEG interferon and direct antiviral agents (DAAs) [10–15].

Although some studies aimed to evaluate HCV Ag testing for diagnosis of HCV and monitor of antiviral therapy, just one systematic review and meta-analysis has been performed to evaluate the utility of HCV Ag for diagnosis [16]. To our knowledge, there has been no study that evaluates simultaneously diagnostic accuracy of HCV Ag to monitor antiviral therapy. We have conducted a systematic review and meta-analysis to evaluate the diagnostic accuracy of HCV Ag testing to identify HCV cases and to monitor antiviral treatment including DAA treatment.

## 2. Materials and Methods

We conducted a systematic review of HCV diagnostic and treatment using HCV Ag test in comparison to HCV RNA detection in accordance with standard PRISMA guidelines (http://www.prisma-statement.org/). Meta-analysis was conducted to evaluate HCV Ag as diagnostic test for HCV infection.

### 2.1. Search Strategy

The studies were identified through a search in PubMed, Lilacs, and Scopus with the following terms: (“hepatitis C” OR “HCV” OR “hepacivirus” OR “hepatitis C virus” AND “HCVAg” OR “hepatitis C core antigen” OR “HCV antigen” AND “diagnosis” OR “detection” OR “laboratory method” AND (“therapy” OR “treatment” OR “antiviral therapy.”). The search strategy has been performed with the inclusion of articles published up to May 30^th^, 2020.

### 2.2. Study Selection

The inclusion and exclusion criteria were established before proceeding to the search and review. The inclusion criteria were as follows: case-control, cross-sectional, cohort, or randomized trial designs; report of HCV Ag detection in serum, plasma, or other biological specimen; report of HCV RNA detection; and studies in English, Portuguese, or Spanish languages. Exclusion criteria were type of article such as editorial comments, reviews, opinion letters, and conference proceedings and studies with insufficient data to estimate the sensitivity and/or specificity of the assay.

The selection of articles for this review study was based carefully on the evaluation of the title and abstract after searching through the keywords, and when an article met the inclusion criteria, the full text was examined and the data extracted.

### 2.3. Data Extraction

Two independent researchers extracted the data and then cross-checked. When the data were unclear or required assumptions, two other researchers were consulted to reach consensus. The values of TP, TN, FP, and FN corresponding to the test evaluated in each article were extracted, and 2 × 2 contingency tables and calculation of sensitivity and specificity were made. The articles that did not have these necessary data were reported explicitly; we contacted the corresponding author and requested this data. Studies whose requested data were not answered were excluded.

### 2.4. Quality Assessment

We used the QUADAS-2 standard (Evaluation of the Quality of Diagnostic accuracy studies 2) to assess the quality and risk of bias in the included studies [17]. This method was designed to evaluate diagnostic accuracy studies through 4 main domains (patient selection, index test, reference standard, and flow and time). Each domain was assessed according to the risk of bias (low, high, or unclear), and in the first three domains, concerns about applicability (low, high, or unclear) were also considered. Two independent reviewers assessed the study's characteristics and methodological quality.

### 2.5. Statistical Analysis

HCV Ag sensitivity was the proportion of samples with a positive HCV RNA test that were also positive on HCV Ag testing. Specificity was the proportion of samples with negative HCV RNA testing that were also negative on HCV Ag testing. Sensitivity and specificity were the primary outcome measures.

Meta-analyses for each HCV Ag test were implemented to calculate the summary statistics, comprising point estimates and their respective 95% confidence intervals (95% CIs) by random-effects model (REM) and study heterogeneity (*I*^2^ statistics). Results from the univariate analyses (including all studies) were compared with the pooled estimates from the bivariate analyses where possible. Descriptive analyses were done for index tests with less than four studies and when substantial heterogeneity was evident from the inspection of the forest and summary plots.

Statistical analyses were performed using program R 4.0.2 with the General Package for Meta-Analysis (Package meta version 4.9-6).

## 3. Results

### 3.1. Study Selection and Characteristics

**Figure 1:**
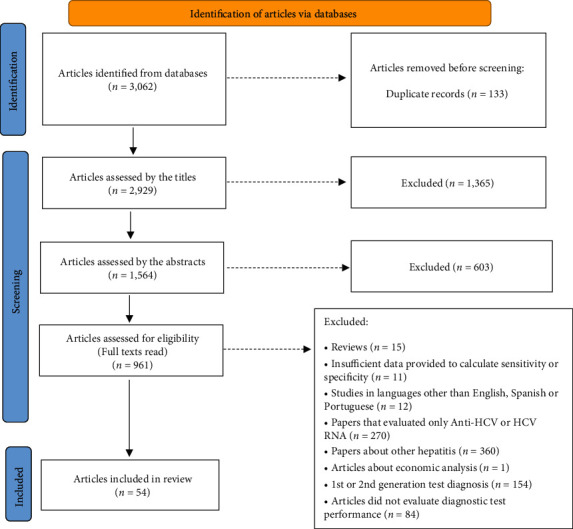
Flow of information and stages of systematic review.

The systematic review identified 3,062 citations, of which 54 papers met inclusion criteria after reading abstract and full text papers (Figure 1). Fifty-four papers used HCV Ag for diagnosis of HCV infection, and some of these studies, as they analyzed different groups, are mentioned more than once in Table 1. And 17 of these 54 studies also provided information on HCV Ag for HCV treatment. Only one study [18] depicted in Table 2 had information about employment of HCV Ag for treatment and not for diagnosis, so this study is not mentioned in Table 1.

Among the 54 studies that evaluated the detection of HCV Ag for the diagnosis of HCV, most of papers (*n* = 42) that used the electrochemiluminescence methodology presented high quality. In general, all assays were qualitative, both EIA or ECLIA.

The main characteristics of studies included here are presented in Tables 1 and 2. Papers from 20 countries were published from 1997 to 2019. A total of 14,286 individuals were included in papers that evaluated HCV Ag for diagnosis, and 15,680 individuals were reported in papers that used HCV Ag to monitor antiviral treatment. A total of 49 studies provided information on HCV genotypes in infected individuals.

### 3.2. Quality Assessment

The overall risk of bias assessment for studies included was evaluated using QUADAS-2 and quality scores of these studies (Supplemental Material, Figures S1, S2, S3). The overall quality of the studies was high where reports using electroquimioluminescence had the best quality. According to figure S2, the risk of bias was higher in the test index.

This was also demonstrated in the individual analysis of the studies (Figure S3). The quality with the ELISA and immunohistochemistry tests, as the index test, proved to be lower [19–23]. And also, two of the three studies evaluated samples of DBS [11, 24].

### 3.3. HCV Antigen for Diagnosis of HCV Active Infection

In this review, different types of studies were included such as the following: longitudinal, transversal, prospective, retrospective, and cohort. One study included HCV/HBV-coinfected individuals, and two studies enrolled HCV/HIV-coinfected patients [25, 26].

Four studies demonstrated sensitivity values lower than 87.2%, but three of these studies employed dried blood spot (DBS) samples for testing [24, 27, 28]. A total of 15 studies used ELISA as detection method where only three of them reported sensitivity ≤ 60%. Only one study used the immunohistochemistry technique to detect HCV Ag [19].  Table 3 demonstrates sensitivity (SE), specificity (SP), positive predictive value (PPV), negative predictive value (NPV), correct classification (accuracy), and Kappa for studies.

Most of studies included genotypes 1a and 1b (29/54), and it was observed a false negative correlation between HCV Ag detection and genotype 3 [29] and high concordance between HCV Ag and HCV RNA results among studies that included HCV patients coinfected with HBV and HIV. Moscato et al. [30] demonstrated a correlation between true positive results and genotype 1b.

**Figure 2:**
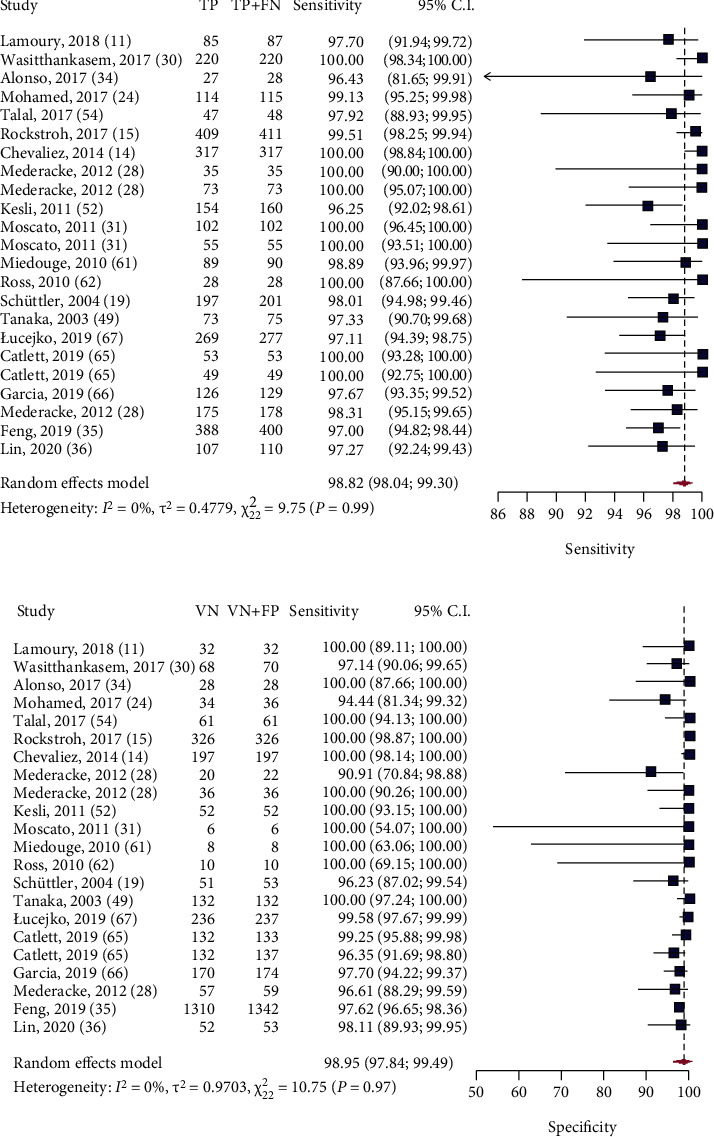
(a) Forest plot of HCV Ag assay sensitivity for the diagnosis of active HCV infection compared to HCV RNA test for all samples regardless of HCV Ab status. TP: true positive; FN: false negative; CI: confidence interval. (b) Forest plot of HCV Ag assay specificity for the diagnosis of active HCV infection compared to HCV RNA test for all samples regardless of HCV Ab status. TP: true positive; FN: false negative; CI: confidence interval.

A total of 23 studies were included in the univariate pooled sensitivity and specificity estimates. The pooled sensitivity and specificity with 95% CI were 98.82% (95%CI = 98.04%; 99.30%) and 98.95% (95%CI = 97.84%; 99.49%), respectively (Figure 2). Heterogeneity is visually assessed in Figures 2(a) and 2(b). The studies appear to be homogeneous in the overall where *I*^2^ was 65% (*P* = 0.97) for specificity and *I*^2^ was 50% (*P* = 0.99) for sensitivity estimates.

### 3.4. HCV Antigen for Monitoring Antiviral Therapy

A total of 18 studies used HCV Ag testing to evaluate antiviral treatment (Table 3). Most of studies (15/18) used ECLIA methodology, and only three of them used ELISA [21, 31, 32]. It was not possible to calculate sensitivity and specificity for most of studies. Nine studies used PEG-IFN/ribavirin as antiviral treatment. Nine out of 18 studies used DAA for antiviral treatment. In addition, only 5 reported concordances between HCV and HCV RNA. Regarding the utility of HCV Ag testing and therapy, five studies demonstrated HCV Ag results at 4 weeks and end of treatment and only two assessed SVR. At the end of treatment, sensitivities varied from 50 to 100% while specificities varied from 92.8% to 100%. At week 4 after beginning of treatment, sensitivities of HCV Ag testing varied from 31.25 to 77.9%. High concordance was found between HCV Ag testing and HCV RNA detection after completion of treatment. Those five studies found agreement from 89.7% to 99.75%.

## 4. Discussion

This review is aimed at evaluating HCV Ag detection test as an alternative to HCV-RNA for the diagnosis of active HCV infection and for the monitoring of antiviral treatment. We concluded that HCV Ag testing can be an alternative to HCV RNA testing for molecular diagnosis of HCV infection.

HCV Ag testing demonstrated pooled sensitivity of 98.82% (95%CI = 98.04%; 99.30%) and specificity of 98.95% (95%CI = 97.84%; 99.49%). ECLIA technology presented higher values of sensitivity and specificity for detecting HCV Ag to identify HCV active cases. However, this technique is more widely used for HCV Ag in comparison to ELISA technique. Galli et al. [33] showed that the ECLIA technique specifically from Abbott Diagnostics Architect HCV Ag, which is the test most commonly used today, detects up to 0.06 pg/ml HCV Ag, whereas older ELISA tests such as Ortho Core antigen detect only samples with 10 pg/ml or more of HCV Ag. Reddy et al. [34] only recruited chronic kidneys and found a sensitivity in HCV Ag detection of only 60% in ELISA tests. It is important to note that this low value may also be related to the clinical condition of these individuals. However, it was not possible to evaluate this information in this review due to few information regarding this topic.

DBS samples were used as biological sample for detecting HCV Ag using ECLIA methodology and showed sensitivity ≤ 87.2% [24, 27, 28] when compared to HCV Ag detection in serum or plasma. Because of this, the use of DBS samples also needs to be further studied to facilitate the diagnosis by detecting HCV Ag. Studies that used serum or plasma with the ECLIA methodology in the diagnosis of HCV Ag showed a sensitivity between 90.4 and 100% compared to HCV RNA detection. Two studies demonstrated lower sensitivities. Florea et al. [35] found 82.4% sensitivity but test demonstrated good performance in patients with HCV RNA ≥ 1000 IU/mL. Fan et al. [36] found 80.2% sensitivity but it was not possible to identify the reasons for this low value.

This study also reviewed several studies that analyzed the use of HCV Ag in monitoring treatment for HCV, with different drugs and at different times of treatment. Most of these studies used ECLIA technique. Gonzalez et al. [21] suggest that ELISA for HCV Ag detection is not used to monitor the end of treatment or an SVR due to the low sensitivity of the test presented in the study (42.5%). Few studies evaluated the concordance between HCV Ag testing and HCV RNA detection to evaluate antiviral treatment, but those studies found values from 89.7% and 95% demonstrating the feasibility of HCV Ag testing to replace HCV RNA detection in low-resource areas. It was also observed that HCV Ag testing has high sensitivity at end of treatment compared to 4 weeks after beginning of treatment. Fan et al. [36] suggested that HCV Ag may be a more sensitive predictor of relapse than HCV RNA after antiviral treatment.

Most of studies that evaluated the accuracy of the HCV Ag test for the diagnosis of HCV were conducted in regions of high HCV prevalence, and this affects the performance of assay to detect HCV acute cases. In the present study, it was observed that sensitivity was not always 100% due to differences in prevalence or population studied. In these cases, such as hemodialysis and people living with HIV, it should be interesting to perform HCV RNA in HCV Ag negative cases to rule out false negative cases [37, 38]. This situation could increase the costs, but it remains a low-cost strategy compared to HCV RNA testing in all subjects. Economic analysis of HCV Ag testing should be relevant in high HCV settings. If the short- and long-term consequences of the HCV Ag test false positives (FPs) and false negatives (FNs) cost less than the extra cost of using the HCV RNA, to use the HCV Ag test will be efficient.

This study presents some limitations such as the evaluation of HIV or HBV coinfection and HCV genotype in meta-analysis results. In addition, it was not possible to determine sensitivity and specificity of HCV Ag testing to evaluate antiviral treatment due to absence of data and few studies have analyzed SVR after treatment with DAAs.

We conclude that HCV Ag detection using ECLIA technique, especially in serum samples, is useful to identify HCV active cases. In addition, this assay presented good results to evaluate antiviral response particularly for PEG-IFN therapy. HCV Ag assay could be an important tool to increase HCV diagnosis in low-resource areas.

## Figures and Tables

**Table 1:** Main characteristic of studies that used HCV Ag testing to evaluate diagnosis of active HCV infection.

Year	Author (reference)	Country	HCV-GT	Study	HCV Ag assay	Gold standard	*N*	Sample	Correlation HCV Ag to HCV RNA	SE (%)	SP (%)
*Chemiluminescence immunoassays*											
2019	Bo Feng et al. [39]	China	1, 2, and 3	Prospective	Architect HCV Ag (Abbott Diagnostics, Wiesbaden Germany)	HCV RNA	782	Serum	NA	90.6%	96.6%
2019	Łucejko et al. [40]	Poland	1	Prospective	Architect HCV Ag (Abbott Diagnostics, Wiesbaden Germany)	HCV RNA	514	Serum	Before *r* = 0.75; during *r* = 0.75; after *r* = 0.15	97.1%	99.6%
2019	Catlett et al. [41]	Australia	NA	Cohort study	Architect HCV Ag (Abbott Diagnostics)	HCV RNA	186	Plasma/DBS	*r* = .77 (plasma) *r* = .82 (DBS)	98.1-100% (plasma), 90.7-92.5 (DBS)	100% (plasma/DBS)
2019	Perez- Garcia et al. [42]	Spain	1a, 1b, 2, 3, and 4	Prospective	Architect HCV Ag (Abbott Diagnostics)	HCV RNA	124	Serum/plasma	*r* = 0.932	97%	95%
2019	Fan et al. [36]	China	1b	Retrospective	Architect HCV Ag (Abbott Diagnostics, Wiesbaden Germany)	COBAS AmpliPrep/COBAS TaqMan HCV test, v2.0 (Roche Molecular Systems, Pleasanton, CA, USA)	135	Serum	*r* = 0.986	80.2%	89.7%
2019	Xiang et al. [43]	China	1b, 2a, 3a, 3b, 6a, 1b/3b	Prospective	Architect HCV Ag (Abbott Diagnostics)	System (Roche Diagnostics)	106	Serum	*r* = 0.894	100%	NA
2018	van Tilborg et al. [44]	Canada	1a, 1b, 1(unspecified), 2, 3, 4, 5, 6	Retrospective	Architect HCV Ag (Abbott Diagnostics)	COBAS AmpliPrep/COBAS TaqMan HCV v2.0 (Roche)	10006	Serum	*r* = 0.97	99.0%	NA
2018	Nguyen et al. [24]	Vietnam	NA	Prospective	Architect HCV Ag (Abbott Diagnostics)	COBAS AmpliPrep/COBAS TaqMan v2 (Roche)	104	DBS	*r* = 0.80	87.2%	100%
2018	Adland et al. [45]	United Kingdom	1, 3	Retrospective	Architect HCV Ag (Abbott Diagnostics)	Abbott RealTime HCV assay (Abbott Molecular, Des Plaines, IL)	305	NA	*r* ^2^ = 0.3	97.7%	NA
2018	Lamoury et al. [28]	Australia	1a, 1b, 2, 3a, 6	Prospective	Architect HCV Ag (Abbott Diagnostics)	COBAS AmpliPrep/COBAS TaqMan HCV test v2.0 (Roche Diagnostics)	120	Plasma/DBS	Plasma *r* = 0.69 DBS *r* = 0.65	91.6% plasma and 82.9% DBS	100% plasma and 96.1% no DBS
2018	Chevaliez et al. [14]	NA	1b	Retrospective	Architect HCV Ag (Abbott Diagnostics)	Roche High-Pure system/COBAS TaqMan HCV test v2.0 (Roche)	631	Plasma	*r* ^2^ = 0.7357	99.80%	NA
2017	Wasitthankasem et al. [29]	Thailand	1a, 1b, 3a, 3b, 6c, 6f, 6i, 6n	Transversal	Architect HCV Ag (Abbott Diagnostics)	Abbott RealTime HCV (Abbott Molecular)	290	Serum	*r* ^2^ = 0.890	100%	97.1%
2017	Mohamed et al. [27]	Tanzania	1a, 4a	Prospective	Architect HCV Ag (Abbott Diagnostics)	COBAS AmpliPrep/COBAS TaqMan HCV test v2.0 (Roche Molecular)	153	Serum/DBS	*r* = 0.80	99.1% serum and 76.7% DBS	94.4% serum and 97.3% DBS
2017	Lucejko et al. [46]	Europe	1a/1b/3a/4-2/29/0/2	Longitudinal	Architect HCV Ag (Abbott Diagnostics)	COBAS AmpliPrep HCV test (Roche Molecular Systems, Pleasanton, CA, USA)	33	Serum/plasma	*r* = 0.625	100%	NA
2017	Talal et al. [47]	EUA	1a, 1b, 2, 3, 4	Prospective	Architect HCV Ag (Abbott Diagnostics)	COBAS TaqMan assay (Roche Diagnostics)	109	Serum	*r* = 0.88	97.9%	100%
2017	Rockstroh et at [15].	NA	1	Retrospective	Architect HCV Ag (Abbott Diagnostics)	Roche High-Pure /COBAS TaqMan HCV test v2.0 (Roche Diagnostics)	737	Plasma	*r* ^2^ = 0.7520	99.05%	100%
2017	Loggi et al. [48]	Italy	1a, 1b, 1(subtype not available), 2, 3, 4	Longitudinal	Architect HCV Ag (Abbott Diagnostics)	COBAS AmpliPrep/COBAS TaqMan HCV test v2.0 (Roche Diagnostics)	96	Serum	*r* = 0.767	100%	NA
2017	Arboledas et al. [49]	Spain	1a, 1b, 2, 3, 4	Prospective	Architect HCV Ag (Abbott Diagnostics)	COBAS AmpliPrep/COBAS TaqMan HCV assay (Roche Diagnostics)	262	Plasma	*r* = 0.83	98.7%	NA
2017	Arboledas et al. [49]	Spain	1a, 1b, 2, 3, 4	Retrospective	Architect HCV Ag (Abbott Diagnostics)	COBAS AmpliPrep/COBAS TaqMan HCV assay (Roche Diagnostics)	132	Plasma	*r* = 0.72	98.5%	NA
2017	Lamoury et al. [11]	Australia	1, 2, 3	Prospective	Architect HCV Ag (Abbott Diagnostics)	COBAS AmpliPrep/COBAS TaqMan HCV test v2.0 (Roche Diagnostics)	92	Plasma	rho = 0.89	94%	NA
2017	Alonso et al. [50]	Spain	1a, 1b, 2c, 3, 4	Retrospective	Architect HCV Ag (Abbott Diagnostics)	COBAS AmpliPrep/COBAS TaqMan HCV assay (Roche Diagnostics)	28	Serum	NA	96.2%	100%
2016	Aghemo et al. [13]	Italy	1, 2, 3, 4, 5	Prospective	Architect HCV Ag (Abbott Diagnostics)	Abbott RealTime HCV (Abbott Molecular)	58	Serum	NA	100%	NA
2016	Pischke et al. [51]	Germany	1, 2, 3, 4	Retrospective longitudinal	Architect HCV Ag (Abbott Diagnostics)	COBAS AmpliPrep/COBAS TaqMan HCV test v2.0 (Roche Diagnostics)	20	NA	NA	100%	NA
2016	Kim et al. [52]	South Korea	1, 2	Retrospective	Architect HCV Ag (Abbott Diagnostics)	COBAS AmpliPrep/COBAS TaqMan HCV assay(Roche Diagnostics)	92	Serum	BG^∗^ *r* = 0.798	98.9%	NA
2015	Kamal et al. [53]	Egypt	4	Prospective longitudinal	Architect HCV Ag (Abbott Diagnostics)	COBAS Amplicor version 2.0 (Roche Molecular)	410	Serum	*r* = 0.944	99.5%	96.8%
2015	Garbuglia et al. [54]	Italy	1a, 1b	Prospective	Architect HCV Ag (Abbott Diagnostics)	Abbott RealTime HCV (Abbott Molecular)	23	Plasma/serum	Day 0: *r* = 0.95 day 1: *r* = 0.79 day 4: *r* = 0.73	100%	NA
2014	Chevaliez et al. [55]	NA	1, 2, 3a, 4, 5a, 6	NA	Architect HCV Ag (Abbott Diagnostics)	COBAS AmpliPrep/COBAS TaqMan HCV test v2.0 (Roche Diagnostics)	514	NA	*r* = 0.89	100%	100%
2014	Garbuglia et al. [56]	Italy	1a, 1b, 1c, 1a/b, 2a, 2c/b, 3a, 4a	Retrospective	Architect HCV Ag (Abbott Diagnostics)	Abbott RealTime HCV (Abbott Molecular)	292	Plasma	*r* = 0.815	90.4%	87.5%
2014	Heidrich et al. [57]	Germany	1n, 2n, 3n	Prospective	Architect HCV Ag (Abbott Diagnostics)	COBAS TaqMan assay v1.0 (Roche Diagnostics)	596	Serum	*r* = 0.803	92.9%	98.9%
2014	Florea et al. [35]	Romania	NA	Retrospective	Architect HCV Ag (Abbott Diagnostics)	COBAS TaqMan assay (Roche Diagnostics)	76	Serum	*r* = 0.980	82.4%	100%
2014	Long et al. [26]	China	1b, 1a	Retrospective longitudinal	Architect HCV Ag (Abbott Diagnostics)	Abbott RealTime HCV (Abbott Molecular)	227	Plasma	HCV: *r* = 0.595 HCV/HIV-1: *r* = 0.884	100%	NA
2013	Hadziyannis et al. [58]	Greece	1, 2, 3, 4	NA	Architect HCV Ag (Abbott Diagnostics)	COBAS Amplicor v2.0 (Roche Molecular)	89	Serum	*r* = 0.89	93.2%	NA
2013	Tedder et al. [10]	UK	1a, 1b, 2, 3	Retrospective	Abbott Architect HCV (Abbott Diagnostics)	RT-PCR TaqMan in-house	54	Plasma	*r* = 0.6	90.7%	NA
2012	Murayama et al. [22]	Japan	1a, 1b, 2a, 2b	Transversal	Architect HCV Ag (Abbott Diagnostics)	COBAS AmpliPrep/COBAS TaqMan (Roche Diagnostics) and Abbott RealTime HCV (Abbott Molecular)	80	Plasma	*r* = 0.9393	100%	NA
2012	Murayama et al. [22]	Japan	1a, 1b, 2a, 2b	Transversal	Lumipulse Ortho HCV Ag (Fujirebio, Tokyo, Japan)	COBAS AmpliPrep/COBAS TaqMan (Roche Diagnostics) and Abbott RealTime HCV(Abbott Molecular)	80	Plasma	*r* = 0.9065	95%	NA
2012	Vermehren et al. [59]	NA	1a, 1b	Prospective	Architect HCV Ag (Abbott Diagnostics)	Abbott RealTime HCV (Abbott Molecular)	160	Serum	(Versant HCV RNA Qualitative Assay) *r* = 0.91 and ART (Abbott RealTime HCV assay) *r* = 092	99.3%	NA
2012	Mederacke et al. [25]	Germany	1a, 3a	Transversal	Architect HCV Ag (Abbott Diagnostics)	COBAS TaqMan assay (Roche Diagnostics)	238	Serum/plasma	HIV/HCV *r* = 0.97 HCV/HBV *r* = 0.04	HCV/HIV: 95.7% HCV/HBV: 100%	HCV/HIV:1100% HCV/HBV:9100%
2011	Kesli et al. [60]	Turkey	1b	Retrospective	Architect HCV Ag (Abbott Diagnostics)	QIAamp viral RNA minikit (Qiagen)	212	Serum	*r* = 0.864	96.3%	100%
2011	Moscato et al. [30]	Italy	1a, 1b, 2a, 2c, 3a, 4	Retrospective	Architect HCV Ag (Abbott Diagnostics)	COBAS TaqMan assay (Roche Diagnostics)	32	Serum	*r* = 0.799	Group A:97.1% group B: 100%	GroupA:100% group B: 100%
2010	Miedouge et al. [61]	France	1, 1a, 1b, 1d, 2, 2a, 2b, 2c, 2k, 2i, 2r, 2x, 3a, 4a, 4c, 4d, 4r, 5a, 6	Cohort	Architect HCV Ag (Abbott Diagnostics)	COBAS TaqMan assay (Roche Diagnostics)	3009	Serum	*r* = 0.904	100%	99.2%
2010	Ross et al. [62]	Germany	1a, 1b, 2a, 3a, 4a, 5a e 6f	Prospective	Architect HCV Ag (Abbott Diagnostics)	Versant HCV RNA v3.0 (Siemens Diagnostics)	394	Serum	*r* = 0.857	100%	100%
2005	Masahiko Takahashi et al. [32]	Japan	NA	Prospective	Lumipulse Ortho HCV Ag (Ortho Clinical Diagnostics)	COBAS Amplicor version 2.0 (Roche Diagnostics)	44	Serum	*r* = 0.870	90.9%	NA
*ELISA*											
2017	Wang et al. [23]	China	NA	Longitudinal	ELISA HCV Ag (LaiBo Biotechnology)	Abbott RealTime HCV (Abbott Molecular)	333	Serum	*r* = 0.891	88.9%	100%
2012	Murayama et al. [22]	Japan	1a, 1b, 2a, 2b	Transversal	ELISA-Ag (Ortho Clinical Diagnostics, Tokyo, Japan)	COBAS AmpliPrep/COBAS TaqMan (Roche Diagnostics) and Abbott RealTime HCV (Abbott Molecular)	80	Plasma	*r* = 0.9666	100%	NA
2012	Murayama et al. [22]	Japan	1a, 1b, 2a, 2b	Transversal	Ortho HCV Ag IRMA (Ortho Clinical Diagnostics, Tokyo, Japan	COBAS AmpliPrep/COBAS TaqMan (Roche Diagnostics) and Abbott RealTime HCV (Abbott Molecular)	80	Plasma	*r* = 0.9666	100%	NA
2012	Murayama et al. [22]	Japan	1a, 1b, 2a, 2b	Transversal	Lumispot Eiken HCV Ag (Eiken Chemical, Tokyo, Japan)	COBAS AmpliPrep/COBAS TaqMan (Roche Diagnostics) and Abbott RealTime HCV (Abbott Molecular)	80	Plasma	*r* = 0.9666	100%	NA
2008	Medhi et al. [63]	India	NA	Transversal	Orthotrak-C™ (Ortho Clinical Diagnostics) (Ortho HCV 3.0)	QIAamp viral RNA minikit (Qiagen) in-house RT-PCR and real-time PCR	250	Serum	NA	96%	100%
2006	Reddy et al. [34]	India	NA	Retrospective	Ortho HCV Ag (Ortho Clinical Diagnostics)	COBAS Amplicor HCV test v2.0 (Roche)	111	Serum	NA	60%	83%
2005	Bouzgarou et al. [64]	Tunisia	1a, 1b, 2c, 3a, 4c/4d	Retrospective	Orthotrak-C™ (Ortho Clinical Diagnostics)	In-house RT-PCR and COBAS Amplicor Monitor v2.0 (Roche version)	76	Serum	NA	84%	89%
2005	González et al. [21]	Spain	1	Cohort	Orthotrak-C™ (Ortho Clinical Diagnostics)	COBAS Amplicor HCV test v2.0 (Roche) and COBAS Amplicor HCV Monitor v2.0; Roche Diagnostics (RNA)	58	Serum	*r* = 0.781	42.5%	100%
2005	Laperche et al. [7]	France	1, 1a, 1b, 2a/c, 3a, 4a, 4c/d	Cohort	Orthotrak-C™ (Ortho Clinical Diagnostics)	COBAS Amplicor HCV test v2.0 (Roche)	35	Plasma	NA	Panel 1 : 100% Panel 2: 96.3%	NA
2005	Massaguer et al. [65]	Spain	1, 2, 3, 4	Cohort	Orthotrak-C™ (Ortho Clinical Diagnostics)	COBAS Amplicor HCV test v2.0 (Roche)	116	Serum	*r* = 0.802	92.1%	100%
2005	Fabrizi et al. [66]	Italy	NA	Prospective	Orthotrak-C™ (Ortho Clinical Diagnostics)	COBAS Amplicor HCV test v2.0 (Roche)	292	Serum	*r* = 0.892	92.7%	97.4%
2004	Soffredini et al. [31]	Italy	1a, 1b, 2a/c, 3a, 4c/d	Prospective	Orthotrak-C™(Ortho Clinical Diagnostics)	Versant HCV RNA v3.0 (Siemens Diagnostics)	111	Serum	*r* = 0.750	94%	NA
2004	Lorenzo et al. [67]	Spain	1a, 1b, 2a/c, 3a	Cohort	Orthotrak-C™ (Ortho Clinical Diagnostics)	COBAS Amplicor HCV test v2.0 (Roche)	16	Serum	NA	87.6%	40%
2004	Schütler et al. [20]	Germany	NA	Prospective	Orthotrak-C™ (Ortho Clinical Diagnostics)	In-house real-time PCR	23	Serum	*r* ^2^ = 0.744	98%	96.2%
2003	Tanaka et al. [68]	Japan	1b, 2a, 2b	Retrospective	In-house	COBAS Amplicor HCV test v1.0 (Roche)	207	Serum	*r* = 0.627	97%	100%
*Immunohistochemistry*											
1997	Ballardini et al. [19]	Italy	1a,1b,2,3a,4a	Retrospective	In-house	RT-PCR in-house	31	Liver tissue	NA	80.6%	NA

Legend: NA: not available; GT: genotype; SE: sensitivity; SP: specificity; V: version; BG^∗^: both genotypes; r and rho: correlation coefficient; r2: coefficient of determination.

**Table 2:** Main characteristic of studies that used HCV Ag testing to evaluate antiviral treatment.

Year	Author	Country	HCV Ag assay	*N*	Treatment	Sensitivity	Specificity	Concordance
2019	Bo Feng et al. [39]	China	Architect HCV Ag (Abbott Diagnostics)	782	PEG-IFN-*α* and ribavirin	NA	NA	NA
2020	Lin et al. [18]		Architect HCV Ag (Abbott Diagnostics)	110	Paritaprevir/ritonavir, ombitasvir e dasabuvir	NA	NA	Baseline: 97.3, week 2 : 54.8, week 4 : 63.9, end: 89.7, post-W12: 98.6
2019	Fan et al. [36]	China	Architect HCV Ag (Abbott Diagnostics)	135	(PEG-INF-*α*) and RBV	Week 12: 96.5%	Week 12: 79.5%	NA
2018	van Tilborg et al. [44]	Canada	Architect HCV Ag (Abbott Diagnostics)	10.006	Treatment of direct acting antivirals (excluding TVR and BOC) with or without PEG-INF, RBV	Week 4: 34.6% End of treatment: 50% Follow-up week 12: 98.2% Follow-up week 24: 100%	Week 4: 86.6% End of treatment: 97.5% Follow-up week 12: 97.7% Follow-up week 24: 100%	NA
2018	Chevaliez et al. [14]	NA	Architect HCV Ag (Abbott Diagnostics)	631	PRV boosted with ritonavir and OBV in a single pill combined with DSV	Week 4: 71.0% Week 12: 96.2% Posttreatment week 12: 87.5%	Week 4: 95.3% Week 12: 97.4% Posttreatment week 12: 98.9%	Concordance: 99.84% Week 4: 97.05% Week 12: 98.8% Posttreatment week 12: 99%
2017	Łucejko et al. [46]	Europe	Architect HCV Ag (Abbott Diagnostics)	33	OBV/PRV/r±DSV±RBV and LDV/SOF	Day 7: 62.5% Week 4: 50%	Day 7: 44.4% Week 4: 81.8% End of treatment: 100%	NA
2017	Rockstroh et al. [15]	NA	Architect HCV Ag (Abbott Diagnostics)	737	RTV-boosted PRV and OBV with DSV coadministered with or without RBV	NA	NA	Concordance between HCV Ag and HCV RNA in week 4 (96.46%) and posttreatment week (99.75%)
2017	Loggi et al. [48]	Italy	Architect HCV Ag (Abbott Diagnostics)	96	3D±RBV; SOF±RBV; SOF+SMV±RBV; SOF+LDV±RBV; SOF+DCV; SOF+PEG-IFN-*α*+RBV	Week 2: 42.8% week 4: 31.25% week 8 and 12: 0%	Week 2: 84.2% Week 4: 78.4% Week 8: 82.1% Week 12: 90.4% Week 16 and 24: 100%	NA
2017	Arboledas et al. [49]	Spain	Architect HCV Ag (Abbott Diagnostics)	262	2D+RBV; 3D±RBV; SOF+DAC±RBV; SOF+LED±RBV; SOF+SIM±RBV; SOF+PEG+RBV; SOF+RBV; SIM+DAC+RBV	NA	NA	Week 1: 56.7% Week 4: 83% End of treatment: 93.5%
2017	Lamoury et al. [11]	Australia	Architect HCV Ag (Abbott Diagnostics)	92	PEG-INF-*α*-2b and RBV	Treatment: 31% Posttreatment: 100% End of treatment: 56% SVR12/24 : 100% sensitivity and specificity	Treatment: 98% Post treatment: 100% End of treatment: 100%	NA
2017	Alonso et al. [50]	Spain	Architect HCV Ag (Abbott Diagnostics)	28	Viekirax+Exviera/Harvoni/Viekirax+Exviera+RBV/Harvoni+RBV/Sovaldi/SOF+DCV/SOF+SMV+RBV	NA	End of treatment: 92.8%	NA
2016	Aghemo et al. [13]	Italy	Architect HCV Ag (Abbott Diagnostics)	58	PRV/RTV/OBV+DSV±RBV or SOF+SMV±RBV/SOF+RBV/PEG-IFN associate SOF+RBV/SOF+RBV or SOF+SMV±RBV	NA	NA	Concordance HCV RNA and HCV Ag: week 2: 40%; week 4: 55%; at the end of treatment, as expected, the agreement between the tests raised to 95%.
2016	Kim et al. [52]	South Korea	Architect HCV Ag (Abbott Diagnostics)	92	PEG-IFN-*α*-2a	Week 4: 33.3%	Week 4: 100%	NA
2015	Kamal et al. [53]	Egypt	Architect HCV Ag (Abbott Diagnostics)	410	PEG-IFN-*α*2a and RBV	Week 4: 100% Week 12: 100%	Week 4: 97.5% Week 12: 99.3%	NA
2014	Florea et al. [35]	Romania	Architect HCV Ag (Abbott Diagnostics)	1782	IFN+RBV	4 weeks: 77.9% 12 weeks: 52.1% 24 weeks: 48.1% 48 weeks: 88.2% 72 week: 96.1%	4 weeks: 100% 12 weeks: 100% 24 weeks: 100% 48 weeks: 100% 72 week: 100%	NA
2005	González et al. [21]	Spain	Orthotrak-C™ (Ortho Clinical Diagnostics)	58	IFN-*α*2*α*+RBV	Week 4: 42.5% Week 12: 10.5% 24 weeks after end of: 88.9%	EOT week 4: 100% EOT week 12: 100% 24 weeks after end of: 100%	NA
2005	Masahiko Takahashi et al. [32]	Japan	Lumipulse Ortho HCV Ag (Ortho Clinical Diagnostics)	44	PEG-IFN-*α*-2a+RBV	Week 7: 76.9% Day 15: 74.1%	NA	Week 7: 79.5%; day 15 = 4: 75%
2004	Soffredini et al. [31]	Italy	Orthotrak-C™ (Ortho Clinical Diagnostics)	111	IFN+RBV	Week 12: 70.5% 6 months after therapy: 94.2%	NA	NA

Legend: PEG: peliguiado; BOC: boceprevir; TVR: telaprevir; DCV: daclastavir; SOF: sofosbuvir; DAC: daclavir; DSV: dasabuvir; LDV: ledipasvir; OBV: ombitasvir; PRV: paritaprevir; RBV: ribavirin; RTV: ritonavir; IFN: interferon; SIM/SMV: simeprevir; NA: not available.

**Table 3:** Sensitivity (SE), specificity (SP), positive predictive value (PPV), negative predictive value (NPV), correct classification (accuracy), and Kappa for studies.

Article	SE	SP	PPV	NPV	Accuracy	Kappa
Lamoury, 2018 [28]	97.7	100.0	100.0	94.1	98.3	97.1
Lamoury, 2018 [28]	88.6	96.9	98.7	75.6	90.8	89.7
Adland, 2018 [45]	94.5	100.0	100.0	90.5	96.4	95.3
Nguyen, 2018 [24]	87.2	100.0	100.0	57.7	89.1	87.8
Wasitthankasem, 2017 [29]	100	97.1	99.1	100.0	99.3	98.0
Alonso, 2017 [50]	96.4	100.0	100.0	96.6	98.2	97.2
Mohamed, 2017 [27]	99.1	94.4	98.3	97.1	98.0	96.7
Mohamed, 2017 [27]	76.7	97.3	98.9	57.1	81.7	80.6
Talal, 2017 [47]	97.9	100.0	100.0	98.4	99.1	98.1
Rockstroh, 2017 [15]	99.5	100.0	100.0	99.4	99.7	98.7
Chevaliez, 2014 [14]	100.0	100.0	100.0	100.0	100.0	98.9
Garbuglia, 2014 [56]	91.4	-	100.0	-	91.4	89.6
Heidrich, 2014 [57]	92.6	98.9	99.6	83.9	94.4	93.2
Mederacke, 2012 [25]	95.7	100.0	100.0	25.0	95.8	93.9
Mederacke, 2012 [25]	100.0	90.9	94.6	100.0	96.5	95.4
Mederacke, 2012 [25]	100.0	100.0	100.0	100.0	100.0	98.9
Yuksel, 2011 [69]	94.3	97.9	99.1	87.0	95.3	94.2
Kesli, 2011 [60]	96.3	100.0	100.0	89.7	97.2	95.9
Moscato, 2011 [30]	100.0	-	97.1	-	97.1	95.2
Moscato, 2011 [30]	100.0	100.0	100.0	100.0	100.0	98.4
Miedouge, 2010 [61]	98.9	100.0	100.0	88.9	99.0	97.3
Ross, 2010 [62]	100.0	100.0	100.0	100.0	100.0	98.8
Medhi, 2008 [63]	96.4	100.0	100.0	86.7	97.1	95.7
Reddy, 2006 [34]	14.3	97.8	60.0	83.0	82.0	80.4
Bouzgarrou, 2005 [64]	83.6	88.9	99.0	29.6	84.0	82.5
Gonzalez, 2005 [21]	68.9	100.0	100.0	74.7	83.8	82.8
Massaguer, 2005 [65]	92.1	100.0	100.0	65.5	93.1	91.7
Fabrizi, 2005 [66]	92.7	97.4	94.7	96.5	95.9	94.8
Lorenzo, 2004 [67]	87.7	40.0	95.9	16.7	84.9	83.2
Schuttler, 2004 [20]	98.0	96.2	99.0	92.7	97.6	96.3
Tanaka, 2000 [68]	97.3	100.0	100.0	98.5	99.0	98.0
Lucejko, 2000 [40]	97.1	99.6	99.6	96.7	98.2	97.2
Catlett, 2019 [41]	100.0	99.2	98.1	100.0	99.5	98.3
Catlett, 2019 [41]	100.0	96.4	90.7	100.0	97.3	96.1
Garcia, 2019 [42]	97.7	97.7	96.9	98.3	97.7	96.7
Mederacke, 2012 [25]	98.3	96.6	98.9	95.0	97.9	96.6
Feng, 2020 [39]	97.0	97.6	92.4	99.1	97.5	96.2
Lin, 2020 [18]	97.3	98.1	99.1	94.5	97.5	96.4
Mean value	95.3			94.0		

## Data Availability

All data are available in the manuscript and supplementary materials.

## Supplementary Materials

Supplementary Materials Supplementary figures demonstrated the quality of studies. S1 shows the risk of bias, S2 shows the applicability of studies, and S3 gives the QUADAS-2 assessment. Figure S1: risk of bias graph. Figure S2: applicability concern graph. Figure S3: QUADAS-2 assessment tool for studies of diagnostic accuracy.
