# Supplementary material for: Performance of HCV Antigen Testing for the Diagnosis and Monitoring of Antiviral Treatment: A Systematic Review and Meta-Analysis
Source: Biomed Res Int. 2022 Jan 4;2022:7348755. doi: 10.1155/2022/7348755 (PMC8752229; doi:10.1155/2022/7348755)
Supplement: Supplementary Materials — Supplementary figures demonstrated the quality of studies. S1 shows the risk of bias, S2 shows the applicability of studies, and S3 gives the QUADAS-2 assessment. Figure S1: risk of bias graph. Figure S2: applicability concern graph. Figure S3: QUADAS-2 assessment tool for studies of diagnostic accuracy. [file 7348755.f1.pdf]

Supplementary figures demonstrated the quality of studies. S1 showed the risk of bias, S2 showed the applicability of studies and S3 gives the QUADAS-2 assessment.

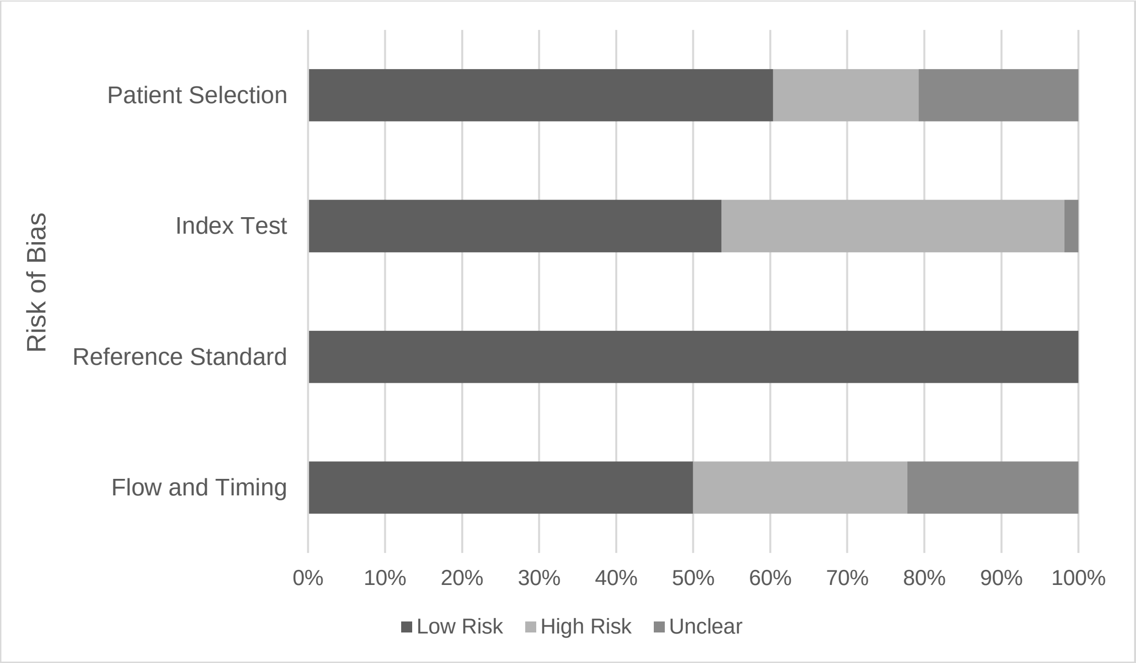


Figure S1. Risk of bias graph

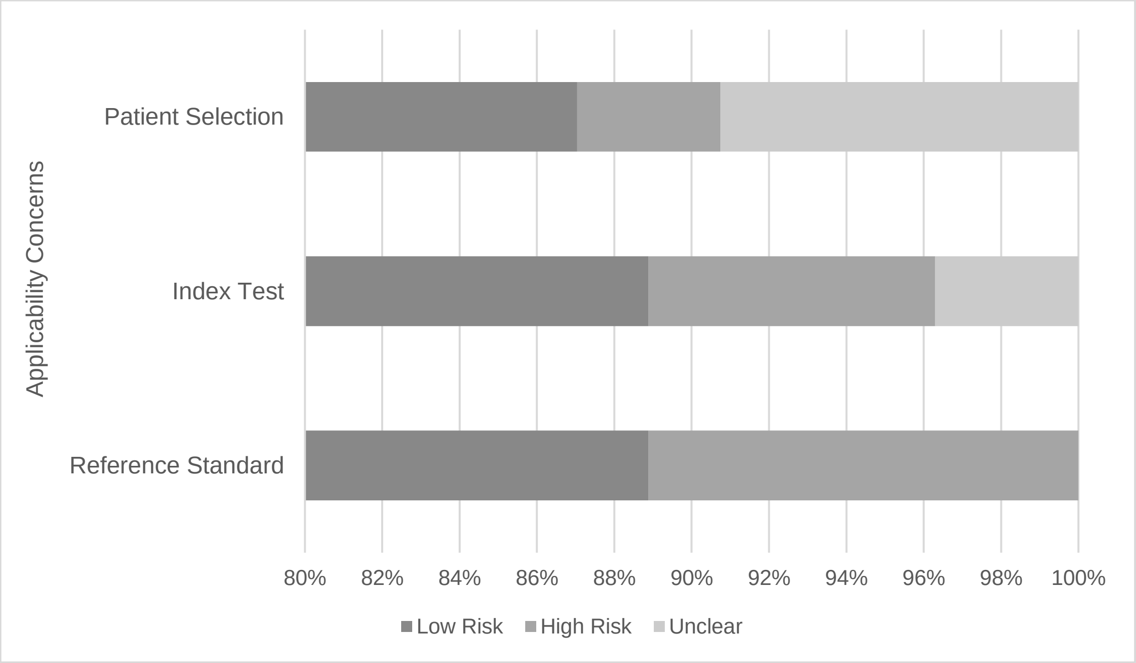


Figure S2. Applicability Concerns graph

Figure S3: QUADAS-2 assessment tool for studies of diagnostic accuracy

| Study | Risk of Bias | | | | Applicability Concerns | | |
|---|---|---|---|---|---|---|---|
| | Patient Selection | Index Test | Reference Standard | Flow and Timing | Patient Selection | Index Test | Reference Standard |
| Bo Feng et al, 2019 (35) | ☹ | ☺ | ☺ | ? | ☺ | ☺ | ☺ |
| Lin et al, 2019 (36) | ☺ | ☺ | ☺ | ☺ | ☺ | ☺ | ☺ |
| Catlett et al, 2019 (65) | ☺ | ☺ | ☺ | ☺ | ☺ | ☺ | ☺ |
| Lucejko et al, 2019 (67) | ☹ | ☺ | ☺ | ☺ | ☺ | ☺ | ☺ |
| Pérez-Garcia et al, 2019 (66) | ☺ | ☹ | ☺ | ? | ☺ | ☺ | ☺ |
| Fan et al, 2019 (37) | ☺ | ☹ | ☺ | ☺ | ☺ | ☺ | ☺ |
| Xiang et al, 2019 (53) | ? | ☹ | ☺ | ☺ | ☺ | ☺ | ☺ |
| Van Tiborg et al, 2018 (38) | ☺ | ☺ | ☺ | ☹ | ☺ | ☺ | ☺ |
| Adland et al, 2018 (44) | ☺ | ☹ | ☺ | ☺ | ☺ | ☺ | ☺ |
| Lamoury et al, 2018 (25) | ? | ☺ | ☺ | ☹ | ☺ | ☺ | ☺ |
| Nguyen et al, 2018 (23) | ☹ | ☹ | ☺ | ? | ☺ | ☺ | ☺ |
| Chevaliez et al, 2018 (14) | ☺ | ☺ | ☺ | ☹ | ? | ☺ | ☺ |
| Wasitthankasim et al, 2017 (30) | ☺ | ☹ | ☺ | ? | ☺ | ☺ | ☺ |
| Loggi et al, 2017 (39) | ☹ | ☹ | ☺ | ☹ | ☺ | ☺ | ☺ |
| Lucejko et al, 2017 (43) | ☺ | ☺ | ☺ | ? | ☺ | ☺ | ☺ |
| Arboledas et al, 2017 (46) | ☺ | ☺ | ☺ | ☺ | ☺ | ☺ | ☺ |
| Lamoury et al, 2017 (11) | ☺ | ☹ | ☺ | ? | ☺ | ☺ | ☺ |
| Wang et al, 2017 (22) | ☺ | ☹ | ☺ | ☺ | ☺ | ☹ | ☺ |
| Alonso et al, 2017 (34) | ☺ | ☺ | ☺ | ☺ | ☺ | ☺ | ☺ |
| Mohamed et al, 2017 (24) | ☺ | ☺ | ☺ | ☹ | ☺ | ☺ | ☹ |
| Talal et al, 2017 (54) | ☺ | ☺ | ☺ | ☺ | ☺ | ☺ | ☺ |
| Rockstroh et al, 2017 (15) | ? | ☹ | ☺ | ☹ | ☺ | ☺ | ☺ |
| Kim et al, 2016 (40) | ? | ☹ | ☺ | ☹ | ☺ | ☺ | ☺ |
| Aghemo et al, 2016 (13) | ☹ | ☹ | ☺ | ☹ | ☺ | ☺ | ☺ |
| Pischke et al, 2016 (56) | ☺ | ☺ | ☺ | ☹ | ☺ | ☺ | ☺ |

| | | | | | | | |
|---|---|---|---|---|---|---|---|
| Garbuglia et al, 2015 (57) | ☺ | ☺ | ☺ | ☺ | ☺ | ☺ | ☺ |
| Kamal et al, 2015 (41) | ☺ | ☹ | ☺ | ☺ | ☺ | ☺ | ☺ |
| Florea et al, 2014 (42) | ☺ | ☺ | ☺ | ☺ | ☺ | ☺ | ☺ |
| Long et al, 2014 (29) | ☺ | ☹ | ☺ | ? | ☺ | ☺ | ☺ |
| Chevaliez et al, 2014 (69) | ☺ | ☺ | ☺ | ☹ | ☺ | ☺ | ☺ |
| Garbuglia et al, 2014 (58) | ☹ | ? | ☺ | ☺ | ☺ | ☺ | ☺ |
| Heidrich et al, 2014 (59) | ☺ | ☺ | ☺ | ☺ | ☺ | ☺ | ☺ |
| Hadziyannis et al, 2013 (60) | ☺ | ☹ | ☺ | ☺ | ☺ | ☺ | ☺ |
| Tedder et al, 2013 (10) | ☹ | ☹ | ☺ | ? | ☺ | ☺ | ☹ |
| Mederacke et al, 2012 (28) | ☺ | ☺ | ☺ | ☹ | ☺ | ☺ | ☹ |
| Murayama et al, 2012 (21) | ☺ | ☹ | ☺ | ☺ | ☺ | ☹ | ☺ |
| Vermehren et al, 2012 (68) | ? | ☺ | ☺ | ☺ | ☺ | ☺ | ☺ |
| Kesli et al, 2011 (52) | ☺ | ☺ | ☺ | ☺ | ☹ | ☺ | ☹ |
| Moscato et al, 2011 (31) | ? | ☺ | ☺ | ☹ | ☺ | ☺ | ☹ |
| Miedouge et al, 2010 (61) | ☺ | ☹ | ☺ | ? | ☺ | ☺ | ☺ |
| Ross et al, 2010 (62) | ? | ☺ | ☺ | ? | ☺ | ☺ | ☺ |
| Medhi et al, 2008 (63) | ☺ | ☺ | ☺ | ☺ | ? | ? | ☺ |
| Reddy et al, 2006 (27) | ? | ☺ | ☺ | ☺ | ☹ | ☹ | ☺ |
| Masahiko Takashi et al, 2005 (33) | ☺ | ☹ | ☺ | ☺ | ☺ | ☺ | ☺ |
| Leperche et al, 2005 (7) | ? | ☺ | ☺ | ☹ | ☹ | ☺ | ☺ |
| Massaguer et al, 2005 (64) | ☹ | ☹ | ☺ | ? | ☺ | ☺ | ☺ |
| Fabrizi et al, 2005 (50) | ☺ | ☺ | ☺ | ? | ☹ | ☺ | ☺ |
| Bouzgarrou et al, 2005 (26) | ? | ☹ | ☺ | ☹ | ☺ | ☺ | ☺ |
| Gonzalez et al, 2005 (20) | ☺ | ☹ | ☺ | ☺ | ☺ | ☺ | ☺ |
| Sofredini et al, 2004 (32) | ? | ☺ | ☺ | ☺ | ☺ | ☺ | ☺ |
| Lorenzo et al, 2004 (51) | ☺ | ☺ | ☺ | ☺ | ☺ | ☺ | ☺ |
| Schuttler et al, 2004 (19) | ☹ | ☹ | ☺ | ☺ | ☺ | ☹ | ☺ |
| Tanaka et al, 2003 (49) | ☺ | ☺ | ☺ | ☹ | ☹ | ? | ☺ |
| Ballardini et al, 1997 (19) | ☹ | ☹ | ☺ | ☺ | ☺ | ☺ | ☹ |

| |
|---|
| |

☺ = Low risk ☹ = High risk ? = Unclear risk
